# Survival benefit of living donor kidney transplantation in patients on hemodialysis

**DOI:** 10.1007/s10157-023-02417-y

**Published:** 2023-10-21

**Authors:** Shunsuke Goto, Hideki Fujii, Makiko Mieno, Takashi Yagisawa, Masanori Abe, Kosaku Nitta, Shinichi Nishi

**Affiliations:** 1 Division of Nephrology and Kidney Center, Kobe University Graduate School of Medicine, 7-5-2 Kusunoki-Cho, Chuo-Ku, Kobe, 650-0017 Japan; 2 Committee of the Renal Data Registry, Japanese Society for Dialysis Therapy, Tokyo, Japan; 3 Center for Information, Jichi Medical University, Tochigi, Japan; 4 Department of Renal Surgery and Transplantation, Jichi Medical University Hospital, Tochigi, Japan; 5 Division of Nephrology, Hypertension, and Endocrinology, Department of Internal Medicine, Nihon University School of Medicine, Tokyo, Japan; 6 Department of Medicine, Kidney Center, Tokyo Women’s Medical University, Tokyo, Japan

**Keywords:** Hemodialysis, Kidney transplantation, Living kidney donor

## Abstract

**Background:**

Donors bravely donate their kidneys because they expect that living donor kidney transplantation (LKT) confers benefits to recipients. However, the magnitude of the survival benefit of LKT is uncertain.

**Methods:**

This prospective cohort study used two Japanese nationwide databases for dialysis and kidney transplantation and included 862 LKT recipients and 285,242 hemodialysis (HD) patients in the main model and 5299 LKT recipients and 151,074 HD patients in the supplementary model. We employed time-dependent model in the main model and assessed the hazard ratio and the difference in the restricted mean survival time (RMST) between LKT recipients and HD patients. In the main analysis of the main model (LKT, *N* = 675; HD, *N* = 675), we matched LKT recipients with HD patients by age, sex, dialysis vintage, and cause of renal failure and excluded HD patients with dementia or performance status grades 2, 3, or 4.

**Results:**

The median observational period was 8.00 (IQR 3.58–8.00) years. LKT was significantly associated with a lower risk of mortality (hazard ratios (95% confidence interval (CI)), 0.50 (0.35–0.72)) and an increase in life expectancy (7-year RMST differences (95% CI), 0.48 (0.35–0.60) years) compared with HD. In subgroup analysis, the survival benefit of LKT was greater in female patients than in male patients in the Cox model; whereas older patients gained longer life expectancy compared with younger patients.

**Conclusions:**

LKT was associated with better survival benefits than HD, and the estimated increase in life expectancy was 0.48 years for 7 years.

**Supplementary Information:**

The online version contains supplementary material available at 10.1007/s10157-023-02417-y.

## Introduction

Living donor kidney transplantation (LKT) has been performed for over six decades [1]. Since LKT requires kidneys from living donors, potential risks for donors cannot be completely eliminated; however, medical professionals make the utmost effort to minimize the physical, psychological, and social risks to individual donors [2, 3]. Nevertheless, living donors donate their kidneys because they expect favorable outcomes for recipients. Therefore, it is important to accurately assess the outcomes of recipients. However, most previous studies have targeted deceased donor kidney transplantation in assessing the survival benefit of kidney transplantation [4], and studies targeting LKTs are scarce. Since the outcomes of LKT recipients could differ from those of recipients of deceased donor kidney transplantation [5, 6], the results of deceased donor kidney transplantation may not be extrapolated to LKT recipients. Although several papers have compared LKT to hemodialysis (HD), most papers targeted a specific population of HD patients or recipients who did not receive modern immunosuppressive agents [7–11]. A study conducted in Denmark, which targeted HD patients registered in the Danish Nephrology Registry, has been reported recently [12]. However, since mortality rates among patients on HD vary across countries [13], these findings may not be generalizable to other countries.

Recently, the restricted mean survival time (RMST) has been used as an alternative measure of treatment effect in some randomized controlled trials with a time-to-event outcome [14]. The RMST is the mean survival time from time 0 to a specific time point. One advantage of the RMST is that it is readily interpretable. Therefore, analyses performed using the RMST may provide LKT donors with additional useful information when they decide to donate their kidneys. Although a recent paper reported the treatment effect of deceased donor kidney transplantation assessed using the RMST [15, 16], to date, no paper has used the RMST for LKT recipients to our knowledge.

This study aimed to assess the survival benefit of LKT in HD patients and determine which subgroup received greater benefit via analyses involving the RMST.

## Methods

### Data sources, study population, and study design

We used the databases of two Japanese nationwide registries, which are the Japanese Society for Dialysis Therapy Renal Data Registry (JRDR) and the Japan Renal Transplantation Registry (JARTRE). Details of the JRDR survey and the JARTRE survey have been described elsewhere [17–21]. Briefly, the Japanese Society for Dialysis Therapy (JSDT) prospectively surveyed the demographic and clinical data of dialysis patients by sending questionnaires to all dialysis facilities in Japan at the end of each year. The response rate exceeded 95% every year. In the JARTRE survey, the Japan Society for Transplantation (JST) and the Japanese Society for Clinical Renal Transplantation (JSCRT) surveyed the demographic and clinical data of kidney transplant recipients and donors in all Japanese kidney transplantation facilities every year. Data from 2009 to 2011 were collected with a flash drive; since 2012, they have been collected online. This survey covers more than 90% of recipients of kidney transplantation after 2008 in Japan. In this survey, we selected HD patients who had the required data at the end of 2009 in the JRDR survey and LKT recipients who received kidney transplantation from 2009 to 2017 in the JARTRE survey.

In this study, we employed the model in which LKT was treated as a time-varying covariate. The time zero was the end of 2009, all patients at time zero were HD patients in the JRDR database, and we matched the data of patients receiving kidney transplantation from 2010 to 2017 in the JRDR database (*N* = 2589) to the data of kidney transplantation recipients in the JARTRE database (*N* = 3317) in this model. The details of method to match patients in the JRDR database to those in the JARTRE database, as follows. First, we created a new identification code based on sex, birth year, year of onset of HD, month of onset of HD, year of reception of kidney transplantation, and month of reception of kidney transplantation. In this method, 99.9% (*N* = 2585) of the subjects in the JRDR database and 99.8% (*N* = 3311) of the subjects in the JARTRE database had unique identification codes. Then, we defined the subjects who had similar identification codes as similar subjects, and 1455 subjects were matched. The other subjects who did not have unique identification codes were matched by defining the subjects who had a similar cause of renal failure as similar subjects, and three subjects were matched. Finally, 1458 subjects were matched.

**Fig. 1:**
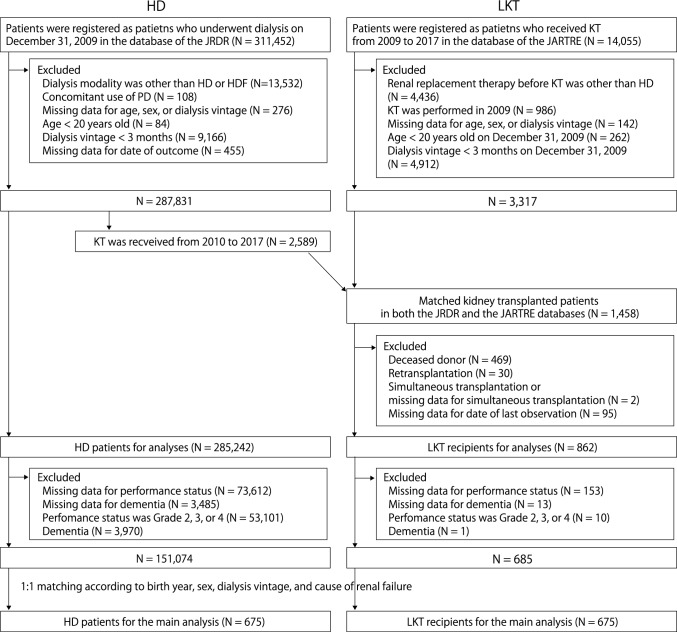
Flow diagram

The patient flowchart is shown in Fig. 1. In the HD group, we enrolled those patients on maintenance HD or hemodiafiltration, whose dialysis vintage lasted three months or longer, and who were aged 20 years or older. We excluded those patients who did not have data on age, sex, or dialysis vintage, those who received combined therapy with peritoneal dialysis, and those whose year of death, kidney transplantation, or dialysis withdrawal was unknown. Then, the patients who received kidney transplantation from 2010 to 2017 were moved to the LKT group.

In the LKT group, we enrolled those patients who received HD before undergoing LKT, those whose dialysis vintage was three months or longer, and those who were aged 20 years or older. We excluded those patients who received LKT in 2009 or did not have data on renal replacement therapy before LKT, age, sex, or dialysis vintage; then, we matched the LKT recipients to the HD patients. After matching, we also excluded those patients who received deceased donor kidney transplantation, kidney re-transplantation, or simultaneous transplantation and did not have data on simultaneous transplantation or date of last observation. Simultaneous transplantation included simultaneous pancreas and kidney transplantation and simultaneous liver and kidney transplantation.

In the main analysis of the main model, we conducted two-step selection to minimize the differences of baseline characteristics. First, we excluded patients whose performance statuses were grades 2, 3, or 4 or who had dementia because most HD patients who received kidney transplantation had a grade 0 or grade 1 performance status and no dementia at baseline (Table 1). Performance status was graded according to the Eastern Cooperative Oncology Group Performance Status classification [22]. Dementia was defined by doctors at each facility. After exclusion, we selected HD patients and LKT recipients matched by birth year, sex, dialysis vintage, and cause of renal failure (glomerulonephritis, diabetes, hypertension, polycystic kidney disease, and other/unknown causes). In the matching, we allowed differences in dialysis vintage of less than 1 year.

**Table 1:** Baseline characteristics before and after matching

	Before matching			After matching		
	LKT (*N* = 862)	HD (*N* = 285,242)	SMD	LKT (*N* = 675)	HD (*N* = 675)	SMD
Age (years)	48.0 ± 12.3	66.8 ± 12.4	1.52	48.3 ± 12.2	48.3 ± 12.2	0.00
Men (%)	59.9	62.0	0.04	60.3	60.3	0.00
Dialysis vintage (years)	3.9 (1.5–8.4)	5.5 (2.5–10.5)	0.32	3.9 (1.5–8.4)	3.9 (1.6–8.4)	0.00
Cause of renal failure (%)						
Glomerulonephritis	48.4	36.6	0.24	49.5	49.5	0.00
DM	18.3	35.9	0.40	18.7	18.7	0.00
Hypertension	4.3	7.3	0.13	4.2	4.2	0.00
Polycystic kidney disease	6.0	3.3	0.13	5.5	5.5	0.00
Others/unknown	23.0	16.9	0.15	22.2	22.2	0.00
Dialysis time (hour/week)	12.0 ± 1.7	11.7 ± 1.8	0.21	12.1 ± 1.7	11.8 ± 1.7	0.14
BMI (kg/m^2^)	21.8 ± 3.5	21.2 ± 4.5	0.06	21.8 ± 3.5	22.0 ± 4.4	0.06
Kt/V	1.38 ± 0.31	1.40 ± 0.30	0.28	1.38 ± 0.32	1.38 ± 0.30	0.01
nPCR (g/kg/day)	0.93 ± 0.18	0.88 ± 0.18	0.14	0.93 ± 0.17	0.91 ± 0.18	0.08
History (%)						
Myocardial infarction	3.3	7.7	0.19	2.9	3.4	0.03
Cerebral hemorrhage	2.2	4.9	0.15	1.7	2.2	0.04
Cerebral infarction	3.6	15.2	0.41	3.2	5.4	0.11
Amputation	0.6	3.1	0.19	0.5	0.5	0.00
Hip fracture	0.6	3.0	0.19	0.5	0.8	0.04
Laboratory test						
Creatinine (mg/dL)	12.5 ± 2.9	10.2 ± 3.0	0.75	12.5 ± 2.9	12.0 ± 2.9	0.18
Hemoglobin (g/dl)	10.8 ± 1.1	10.6 ± 1.3	0.21	10.8 ± 1.1	10.7 ± 1.3	0.07
Albumin (g/dL)	3.9 ± 0.4	3.7 ± 0.4	0.59	3.9 ± 0.3	3.9 ± 0.4	0.00
Corrected calcium (mg/dL)	9.3 ± 0.8	9.3 ± 0.9	0.02	9.3 ± 0.9	9.3 ± 0.8	0.02
Phosphorus (mg/dL)	5.6 ± 1.6	5.1 ± 1.5	0.35	5.6 ± 1.6	5.5 ± 1.5	0.07
Intact PTH (pg/mL)	131 (73–223)	124 (63–208)	0.11	131 (73–218)	132 (67–226)	0.03
CRP (mg/dL)	0.1 (0.0–0.2)	0.1 (0.0–0.4)	0.12	0.1 (0.0–0.2)	0.1 (0.0–0.2)	0.02
Performance status (%)						
Grade 0	75.9	44.7	0.67	76.9	73.2	0.09
Grade 1	22.7	29.4	0.15	23.1	26.8	0.09
Grade 2	1.0	13.0	0.48	0.0	0.0	0.00
Grade 3	0.4	7.1	0.36	0.0	0.0	0.00
Grade 4	0.0	5.8	0.35	0.0	0.0	0.00
Dementia (%)						
No dementia	99.7	89.8	0.46	100.0	100.0	0.00
Dementia not requiring care	0.3	4.5	0.28	0.0	0.0	0.00
Dementia requiring care	0.0	5.7	0.35	0.0	0.0	0.00

*LKT* living kidney transplantation; *HD* hemodialysis; *SMD* standardized mean difference; *DM* diabetes mellitus; *BMI* body mass index; *nPCR* normalized protein catabolic rate; *PTH* parathyroid hormone; *CRP* C-reactive protein

All patients were followed up until death, the end of the study (December 31, 2017), or loss to follow-up. Patients without death were censored at the end of the study or loss to follow-up. Since the month of the start of dialysis, death, or loss to follow-up was unknown for some HD patients, we assigned the month of June in such cases for these patients. Since there were no data on the day of death or loss to follow-up in the HD group (though the LKT group had the required data), we defined the day of death or loss to follow-up in the HD group as the midpoint of the days in the month of the death or loss to follow-up. The study protocol was approved by the Ethics Committee of the JSDT (approval number: 49), the Ethics Committee at Kobe University Graduate School of Medicine (approval number: B200241), and the ethics committee of the JST, and was conducted per the principles of the Helsinki Declaration. The need for participants’ informed consent was waived. The studies were in accordance the Declaration of Istanbul.

### Statistical analysis

Data are presented as the mean and standard deviation for continuous variables with a normal distribution, the median and interquartile range for continuous variables with a skewed distribution, or the number and percentage for categorical variables.

Survival curves in each group were drawn using the Simon and Makuch method [23] with statistical comparison using the Mantel–Byar method [24, 25]. The time-dependent Cox proportional hazard regression analysis was also used to compare mortality rates between groups. In addition, we assessed differences in the RMST and ratios of the restricted mean time lost (RMTL), which is the area above the survival curve [26], between the HD and LKT groups. The following three time points were used for this analysis: 3 years, 5 years, and 7 years.

Furthermore, we performed similar analysis in the model to simply compare LKT recipients in the JARTRE database to HD patients in the JRDR database as a supplementary model having larger sample size compared with the main model. In this model, the time zero in the HD group was the end of 2009, whereas the time zero in the LKT group was the moment LKT was performed. The patient flow chart in the model is shown in Supplementary Fig. 1. In main analysis of this model, we targeted patients who received LKT from 2009 to 2013; however, we also performed the analysis targeting LKT recipients from 2009 to 2010 or those from 2009 to 2017 as sensitivity analysis. In this model, survival curves were drawn using Kaplan–Meier methods with statistical comparison using the log-rank test.

We also performed other sensitivity analyses. First, we performed a conventional multivariable Cox proportional hazard regression analysis in the unmatched population. In this analysis, the adjusted variables were all the baseline characteristics listed in Table 1. If serum albumin levels were under 4 g/dL, serum calcium levels were corrected using the following formula: corrected calcium = serum calcium (mg/dL) + [4 − serum albumin (g/dL)]. If the parathyroid hormone (PTH) assay was whole, whole PTH levels were multiplied by 1.7 to obtain equivalent values measured by an intact PTH assay [27]. Dialysis vintage, intact PTH, and C-reactive protein (CRP) were log-transformed to normalize the distribution. Second, we also performed a conventional multivariable Cox proportional hazard regression analysis after multiple imputations were conducted because some covariates had missing data (Supplementary Table 1). We imputed the missing values using fully conditional chained equations, created five imputed datasets, and combined them using Rubin's rules [28]. Third, propensity score matching was performed using nearest-neighbor 1:1 matching. Propensity scores were created using multivariable logistic regression analyses to estimate the probability of receiving or not receiving LKT. All variables using a conventional multivariable Cox proportional hazard regression analysis were included as covariates. Fourth, the inverse probability of treatment weighting (IPTW) was used to balance differences across groups [29]. We performed a Cox proportional hazard regression analysis after each patient was weighted by the inverse of the probability of that patient being assigned to LKT. Fifth, we performed a Cox proportional hazard regression analysis for only HD patients with an HD frequency of three times per week or an HD frequency of three times per week and ≥ 4-h session length. Finally, in the supplementary model, we performed a Cox proportional hazard regression analysis for only HD patients with a grade 0 performance status and a hazard ratio of death was estimated for each of the two intervals of time following the baseline: less than 1 year and at least 1 year.

Subgroup analyses were performed for age, sex, dialysis vintage, and causes of renal failure in a Cox proportional hazard regression analysis and the assessment of differences in the RMST and ratios of the RMTL. In the assessment of differences in the RMST and ratios of the RMTL, interaction terms were estimated using Tian et al.’s method [30, 31].

In the comparison of each baseline variable, a standardized mean difference of < 10% was considered to denote a negligible difference between the groups. A* p* value of < 0.05 was considered statistically significant. All statistical analyses were performed using Stata/MP 14.2 software for Windows (Stata, College Station, TX, USA).

## Results

**Fig. 2:**
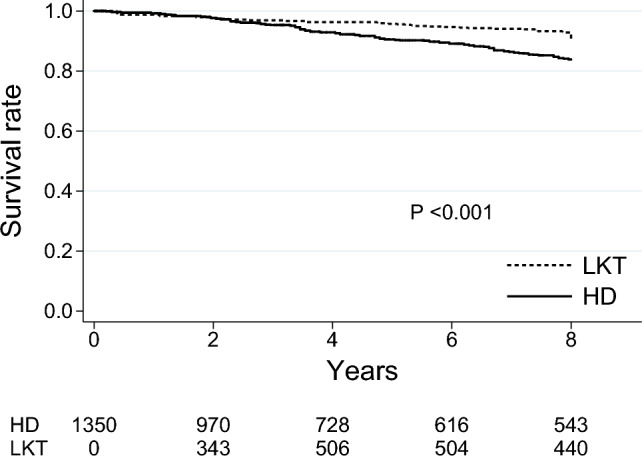
Survival curves using the Simon and Makuch method in the matched cohort

Table 1 shows the baseline characteristics for each group before and after matching and restriction in the main model. Most variables were balanced after matching and restriction, except for the dialysis time, history of cerebral infarction, and serum creatinine levels. The median observational periods were 8.00 (IQR 3.58–8.00) years. The survival curve using the Simon and Makuch method showed that the survival rate in the LKT group was significantly higher than that in the HD group (Fig. 2). The annual mortality rates for HD patients and LKT recipients were 2.03 and 1.20 per 100 patient-year, respectively. At 5 years, patient survival in the HD and the LKT groups was 87.2% and 98.0%, respectively.

**Fig. 3:**
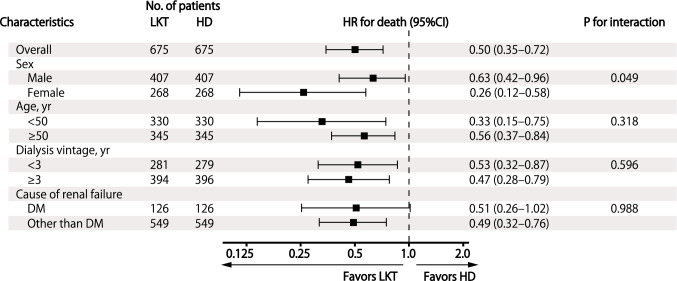
Subgroup analysis for the association with all-cause mortality

Figure 3 shows the results of the Cox proportion hazard regression analyses in all patients and among the various subgroups. LKT was significantly associated with a lower risk of mortality than HD (hazard ratio (95% confidence interval (CI)), 0.50 (0.35–0.72)). In the sensitivity analyses, the association was consistent (Table 2). In most subgroups, LKT was significantly associated with a lower risk of mortality than HD. The survival benefit of LKT vs. HD was more pronounced in female patients; whereas, the benefit did not differ significantly regarding of age, dialysis vintage, or diabetic nephropathy.

**Table 2:** Cox proportional hazard models for all-cause mortality

	Number of patients		HR (95% CI)
	LKT	HD	
Main analysis	675	675	0.50 (0.35–0.72)
Only patients with three times per week of HD frequently	649	649	0.51 (0.36–0.74)
Only patients with three times per week of HD frequently and ≥ 4-h session length	546	546	0.48 (0.32–0.73)
Propensity score matching	438	438	0.58 (0.36–0.92)
IPTW	440	125,224	0.31 (0.16–0.59)
Multivariate Cox proportional hazard model	440	125,224	0.46 (0.31–0.68)
Multivariate Cox proportional hazard model after multiple imputations	862	285,242	0.44 (0.34–0.58)

*HR* hazard ratio; *LKT* living donor kidney transplantation; *HD* hemodialysis; *IPTW* inverse probability of treatment weighting

Table 3 and Supplementary Tables 2–3 show the RMST and the RMTL in the LKT group and the HD group. The 7-year RMST in the LKT group was significantly higher than that in the HD group (LKT vs. HD; 6.90 (6.85–6.95) vs. 6.42 (6.31–6.54) years). The 7-year RMST differences were 0.48 (0.35–0.60) years. In a subgroup analysis using RMST, the survival benefit of LKT vs. HD was more pronounced in older patients; whereas, the benefit did not differ significantly among subgroups stratified by sex, dialysis vintage, or diabetic nephropathy. The results of the RMTL ratio among subgroups had trends similar to those in Cox proportional hazard regression analyses among subgroups.

**Table 3:** The 7-year RMST differences and RMTL ratios in all patients and subgroups

	RMST (years)		Differences in RMST (years)	*P* for interaction	RMTL ratio	*P* for interaction
	LKT	HD				
Overall	6.90 (6.85–6.95)	6.42 (6.31–6.54)	0.48 (0.35–0.60)		0.18 (0.11–0.29)	
Sex						
Male	6.86 (6.79–6.94)	6.37 (6.22–6.52)	0.49 (0.32–0.66)	0.601	0.22 (0.12–0.39)	0.182
Female	6.95 (6.91–7.00)	6.50 (6.33–6.67)	0.45 (0.27–0.63)		0.09 (0.03–0.28)	
Age, yr						
< 50	6.97 (6.94–7.01)	6.68 (6.56–6.81)	0.29 (0.16–0.42)	0.001	0.09 (0.02–0.31)	0.221
> 50	6.83 (6.74–6.92)	6.17 (5.99–6.36)	0.65 (0.44–0.86)		0.21 (0.12–0.37)	
Dialysis vintage, yr						
< 3	6.93 (6.88–6.98)	6.37 (6.18–6.55)	0.56 (0.37–0.75)	0.319	0.11 (0.05–0.24)	0.297
> 3	6.88 (6.80–6.95)	6.46 (6.32–6.61)	0.42 (0.25–0.58)		0.23 (0.12–0.44)	
Cause of renal failure						
DM	6.82 (6.65–6.98)	6.15 (5.83–6.46)	0.67 (0.32–1.03)	0.135	0.21 (0.08–0.56)	0.593
Other than DM	6.92 (6.87–6.96)	6.49 (6.36–6.61)	0.43 (0.30–0.56)		0.16 (0.09–0.30)	

*RMST* restricted mean survival time; *RMTL* restricted mean time lost; *LKT* living donor kidney transplantation; *HD* hemodialysis; *DM* diabetes mellitus

Supplementary Table 4 shows the baseline characteristics for each group before and after matching and restriction in the supplementary model to simply compare LKT recipients to HD patients. Variables in the HD group of this model after matching and restriction did not differ significantly from those in the HD group of the main model. The median observational period was 7.87 (interquartile range (IQR) 3.20–8.00) years. In this model, the annual mortality rates for HD patients and LKT recipients were 2.72 and 1.02 per 100 patient-year, respectively. At 5 years, patient survival in the HD and the LKT groups was 87.8% and 95.9%, respectively. The Kaplan–Meier survival curve showed that the survival rate in the LKT group was significantly higher than that in the HD group (Supplementary Fig. 2). The association was consistent in Cox proportional hazard regression analyses including sensitivity analysis (Supplementary Table 5). Although the hazard ratio during the first year after the baseline was higher than that at 1 year or longer after the baseline, mortality in the LKT group was significantly lower than that in the HD group during even the first year (hazard ratio (95% CI), the first 1 year, 0.60 (0.39–0.91) and, 1 year or longer, 0.35 (0.29–0.43), respectively). In subgroup analysis using Cox proportional hazard models, the survival benefit of LKT vs. HD was more pronounced in younger patients and patients with shorter dialysis vintage in addition to female patients (Supplementary Fig. 3). In subgroup analysis using RMST, the survival benefit of LKT vs. HD was more pronounced in patients with diabetic nephropathy in addition to older patients (Supplementary Table 6–8).

## Discussion

In this study, LKT was associated with a 50% reduction in mortality. A recent meta-analysis has shown that kidney transplantation was associated with a 55% reduction in mortality [4]. Although the patients in most of the populations included in the meta-analysis received deceased donor kidney transplantation, one study has reported that LKT was associated with a 70% mortality risk reduction [12]. In our study, LKT was associated with a 50% reduction in mortality, which was similar to benefit of deceased donor kidney transplantation and a little smaller than that of LKT reported in previous papers [4, 12]. In addition, our study demonstrated that annual mortality rates were 2.03 per 100 patient-years and 5-year survival rates were 87.2% in the HD group, which was better than those of HD patients on waiting lists in previous studies [12, 32, 33]. Taken together, LKT may have a significant survival benefit even in patients on HD with low mortality rates.

We assessed the survival benefit of LKT using not only the hazard ratio but also RMST. The analysis using RMST revealed that LKT was associated with an increase in life expectancy of 0.48 years during an observation period of 7 years and 0.25 years during an observation period of 5 years. Previous studies have shown that deceased donor kidney transplantation has been associated with an increase in life expectancy of 0.53 years or 0.20 years over 5 years [15, 16], the survival benefit of LKT in our study may be similar or a little smaller than that of the deceased donor kidney transplantation in terms of the RMST. The discrepancy with the results in the analysis performed using the hazard ratio may be due to the good survival of HD patients in our study. When hazard ratios are similar, the difference of RMST reduces in patients with better survival rates. Since the survival in our study was better than that reported in previous studies [15, 16], the RMST in our study could be smaller than that in previous studies.

RMST has been recently used in the assessment of survival benefits. It may be difficult for patients and clinicians to understand the exact meaning of the hazard ratio; meanwhile, the difference in life expectancy as assessed by RMST is simple and understandable [15]. Since understandability is important for people who will donate their living kidneys, the analysis by RMST should be performed in the assessment of the survival benefit of LKT.

Per our findings, kidney transplantation provided greater survival benefits in older patients in the analysis using RMST. Since this finding is in line with those of a previous study using RMST [16], older patients may gain longer life expectancy compared to younger patients. On the other hand, younger patients may have a lower hazard ratio than older patients per the results of the analysis performed using Cox models. Since previous studies have shown that hazard ratios in younger patients were lower or equal to those in older patients [12, 33–37], this finding about the hazard ratio is also in line with the results of previous studies. The results assessed by RMST may sometimes be inconsistent with those obtained using the hazard ratio [38]. However, since studies that used both RMST and the hazard ratio are scarce in subgroup analyses for the survival benefit of kidney transplantation, further studies need to be conducted to pinpoint the reason for the discrepancy.

The survival benefit of LKT was greater in female patients than in male patients in the Cox model; however, the benefit did not differ significantly between the two sexes in the analysis using RMST. The results of females' greater benefit were inconsistent with those of previous studies, which have shown that the survival benefit in female patients was similar to that in male patients [33, 34, 36, 37]. This discrepancy could be due to the difference in survival rate between female patients and male patients. The survival rate did not differ significantly between the two sexes in the aforementioned studies [33, 34, 36, 37], while female patients had a significantly better survival rate than male patients in our study. When the gain in life expectancy does not differ significantly, the Cox model shows a lower hazard ratio in patients with better survival rates. Therefore, the increase in life expectancy may not differ significantly between female patients and male patients.

In our study, patients with diabetic nephropathy may gain longer life expectancy than those without diabetic nephropathy. To the best of our knowledge, no paper has compared RMSTs between patients with and without diabetic nephropathy. Previous studies using hazard ratios have reported inconsistent results [32–34, 36, 37]. As we mentioned above, the difference of RMST reduces in patients with better survival rates when hazard ratio does not differ significantly. Since HD patients with diabetic nephropathy have a poor prognosis [39, 40], the gained life expectancy of patients with diabetic nephropathy might be longer than that of those without diabetic nephropathy.

Our study had several limitations. First, we could not use the data of a transplant waiting list. In an assessment of the survival benefit of kidney transplantation, many papers have compared dialysis patients on a waiting list because many dialysis patients have contraindications to kidney transplantation, and not using patients on a waiting list introduces a significant selection bias [4]. Our approach of using the data of the performance and dementia statuses could minimize the selection bias because the survival rate in the HD group of our study was better than the rates in previous studies using waiting lists [12, 15, 16, 32, 33]. However, in our study, patients in the HD group had higher proportion of cerebral infarction, shorter dialysis time, and lower serum creatinine levels compared with those in the LKT group, though we performed multivariable Cox proportional hazard model adjusting these factors and the results were comparable with that in the main analysis. On the other hand, some characteristics may differ between patients receiving LKT and patients receiving deceased donor kidney transplantation. In Japan, dialysis vintage differed significantly (LKT vs. deceased donor kidney transplantation, 3.0 vs. 15.8 years in 2015) [20]. Therefore, even if HD patients on waiting lists were enrolled, the selection bias could not have been sufficiently minimized. Further studies are needed to clarify whether matching using performance status and dementia is appropriate for comparison between LKT and HD. Second, unique identifying variables to match patients in the two databases were not lacking. However, the identification code created in our study was unique for over 99% of patients, and this limitation is unlikely to render the treatment effect systematically biased in favor of either transplantation or dialysis [32]. Third, since the population in our study had good survival rate, generalizability to the population with poor survival rate was uncertain. Fourth, the data about history of malignancy is lacking in this study. Since Kidney Disease: Improving Global Outcomes guidelines recommend that patients with active malignancy be excluded from kidney transplantation [41], the number of patients with history of malignancy in LKT group will be small. Further studies are needed to clarify how this lack of data influences the benefit of LKT.

In conclusion, LKT was associated with better survival benefits than HD, and the estimated increase in life expectancy was 0.48 years for 7 years. Although the benefit was observed across most subgroups, the magnitude of the benefit may differ among subgroups.

### Supplementary Information

Below is the link to the electronic supplementary material.

Supplementary file1 (PDF 694 KB)

## Data Availability

The data underlying this article were provided by the JSDT, JST, and JSCRT by permission. Data will be shared on request to the corresponding author with permission of the JSDT, JST, and JSCRT.
