# Supplementary material for: Survival benefit of living donor kidney transplantation in patients on hemodialysis
Source: Clin Exp Nephrol. 2023 Oct 21;28(2):165–74. doi: 10.1007/s10157-023-02417-y (PMC10808530; doi:10.1007/s10157-023-02417-y)
Supplement: Supplementary file 1 — Supplementary file1 (PDF 694 KB) [file 10157_2023_2417_MOESM1_ESM.pdf]

Supplementary materials

Survival benefit of living donor kidney transplantation in patients on hemodialysis

Supplementary Figure 1. Flow diagram in the supplementary model.

Supplementary Figure 2. Kaplan–Meier cumulative survival rate in the matched cohort in the supplementary model.

Supplementary Figure 3. Subgroup analysis for the association with all-cause mortality in the supplementary model.

Supplementary Table 1. Number of missing values of each variable.

Supplementary Table 2. The five-year RMST differences and RMTL ratios in all patients and subgroups.

Supplementary Table 3. The three-year RMST differences and RMTL ratios in all patients and subgroups.

Supplementary Table 4. Baseline characteristics before and after matching in the supplementary model.

Supplementary Table 5. Sensitivity analysis in the supplementary model.

Supplementary Table 6. The seven-year RMST differences and RMTL ratios in all patients and subgroups in the supplementary model.

Supplementary Table 7. The five-year RMST differences and RMTL ratios in all patients and subgroups in the supplementary model.

Supplementary Table 8. The three-year RMST differences and RMTL ratios in all patients and subgroups in the supplementary model.

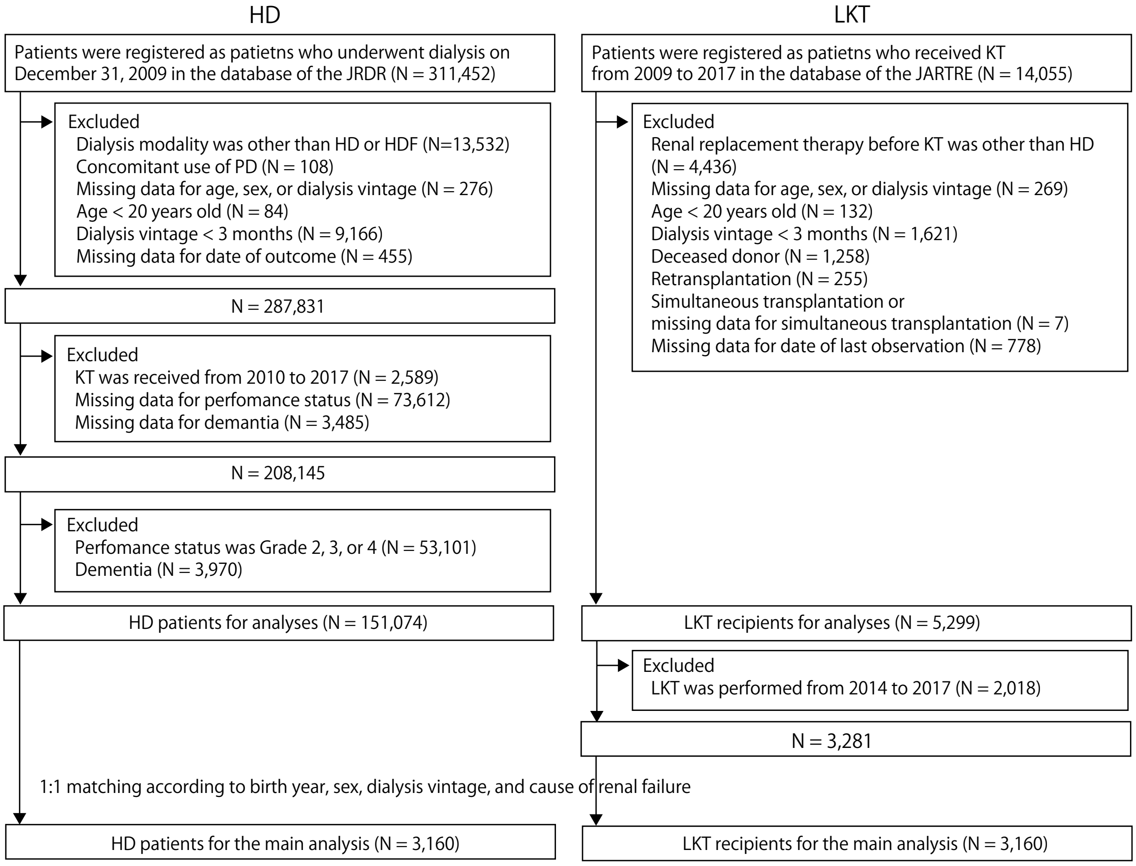

Supplementary Figure 1. Flow diagram in the supplementary model.

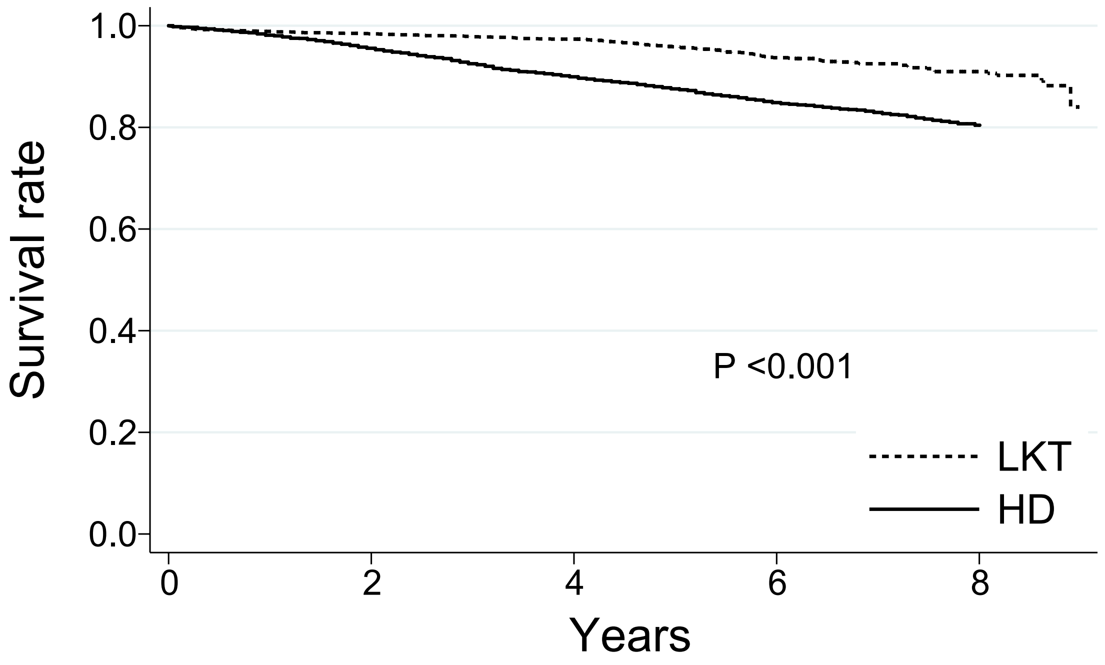

HD	3160	3014	2834	2673	2532
LKT	3160	2968	2516	1138	288

Supplementary Figure 2. Kaplan–Meier cumulative survival rate in the matched cohort in the supplementary model.

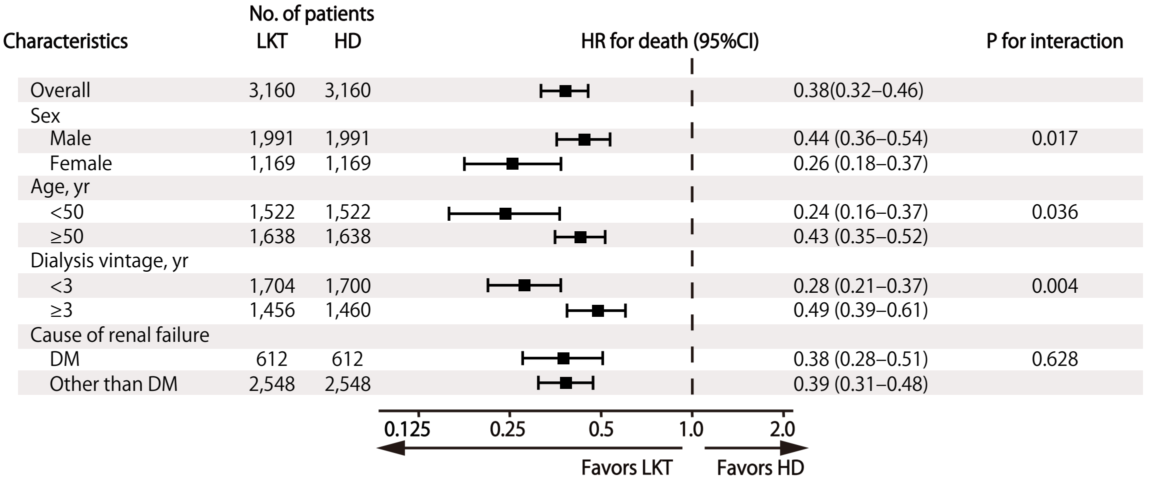

Supplementary Figure 3. Subgroup analysis for the association with all-cause mortality in the supplementary model.

Supplementary Table 1. Number of missing values of each variable.

	No. of missing value (%)			
	Model 1		Model 2	
	LKT (N = 5,299)	HD (N = 208,145)	LKT (N = 862)	HD (N = 285,242)
Age	0	0	0	0
Gender	0	0	0	0
Dialysis vintage	0	0	0	0
Cause of renal failure	0	0	0	0
Dialysis time		2,116 (1.0)	84 (9.7)	46,760 (16.4)
BMI		27,649 (13.3)	176 (20.4)	86,240 (30.2)
Kt/V		12,699 (6.1)	131 (15.2)	66,650 (23.4)
nPCR		12,410 (6.0)	129 (15.0)	66,322 (23.3)
Past history of myocardial infarction		7,697 (3.7)	165 (19.1)	78,277 (27.4)
Past history of cerebral hemorrhage		7,741 (3.7)	165 (19.1)	78,628 (27.6)
Past history of cerebral infarction		7,681 (3.7)	169 (19.6)	78,594 (27.6)
Past history of amputation		6,761 (3.2)	158 (18.3)	76,030 (26.3)
Past history of hip fracture		7,935 (3.8)	167 (19.4)	78,826 (27.6)
Creatinine		1,604 (0.8)	81 (9.4)	51,172 (17.9)
Hemoglobin		2,930 (1.4)	88 (10.2)	53,097 (18.6)
Albumin		7,040 (3.4)	98 (11.4)	57,968 (20.3)
Corrected calcium		2,015 (1.0)	81 (9.4)	51,594 (18.1)
Phosphorus		2,403 (1.2)	84 (9.7)	52,219 (18.3)
Intact PTH		15,980 (7.7)	158 (18.3)	76,776 (26.9)
CRP		44,001 (21.1)	234 (27.1)	101,596 (35.6)
Performance Status		0	153 (17.7)	73,612 (25.8)
Dementia		0	150 (17.4)	72,603 (25.5)

Abbreviations: LKT, living donor kidney transplantation; HD, hemodialysis; BMI, body mass index; nPCR, normalized protein catabolic rate; PTH, parathyroid hormone; CRP, C-reactive protein.

Supplementary Table 2. The five-year RMST differences and RMTL ratios in all patients and subgroups.

	RMST (years)		Difference in RMST (years)	P for interaction	Ratio of RMTL	P for interaction
	LKT	HD				
Overall	4.96 (4.93–4.99)	4.71 (4.64–4.78)	0.25 (0.18–0.44)		0.15 (0.07–0.29)	
Sex						
Male	4.94 (4.90–4.98)	4.69 (4.59–4.78)	0.26 (0.15–0.46)	0.646	0.19 (0.09–0.41)	0.256
Female	4.98 (4.96–5.01)	4.75 (4.64–4.85)	0.24 (0.13–0.47)		0.07 (0.01–0.30)	
Age, yr						
<50	4.99 (4.98–5.01)	4.86 (4.79–4.93)	0.13 (0.06–0.30)	<0.001	0.06 (0.01–0.38)	0.341
>50	4.93 (4.87–4.98)	4.57 (4.45–4.68)	0.36 (0.23–0.62)		0.17 (0.08–0.36)	
Dialysis vintage, yr						
<3	4.99 (4.97–5.00)	4.68 (4.57–4.79)	0.31 (0.19–0.49)	0.281	0.04 (0.01–0.16)	0.047
>3	4.94 (4.89–4.98)	4.73 (4.65–4.81)	0.21 (0.11–0.45)		0.23 (0.11–0.51)	
Cause of renal failure						
DM	4.90 (4.80–5.00)	4.57 (4.38–4.75)	0.34 (0.12–0.83)	0.228	0.23 (0.08–0.68)	0.289
Other than DM	4.97 (4.95–5.00)	4.74 (4.67–4.82)	0.23 (0.15–0.38)		0.12 (0.05–0.28)	

Abbreviation: RMST, restricted mean survival time; RMTL, restricted mean time lost; LKT, living donor kidney transplantation; HD, hemodialysis; DM, diabetes mellitus.

Supplementary Table 3. The three-year RMST differences and RMTL ratios in all patients and subgroups.

	RMST (years)		Difference in RMST (years)	P for interaction	Ratio of RMTL	P for interaction
	LKT	HD				
Overall	2.99 (2.98–3.00)	2.91 (2.88–2.94)	0.08 (0.05–0.11)		0.15 (0.06–0.36)	
Sex						
Male	2.98 (2.96–3.00)	2.90 (2.86–2.94)	0.08 (0.03–0.12)	0.803	0.20 (0.07–0.55)	0.214
Female	3.00 (2.99–3.00)	2.92 (2.87–2.97)	0.08 (0.03–0.12)		0.04 (0.01–0.35)	
Age, yr						
<50	3.00 (3.00–3.00)	2.97 (2.94–3.00)	0.03 (0.00–0.06)	0.001	0.04 (0.00–0.37)	0.274
>50	2.98 (2.95–3.00)	2.85 (2.80–2.91)	0.12 (0.06–0.18)		0.17 (0.07–0.44)	
Dialysis vintage, yr						
<3	3.00 (3.00–3.00)	2.90 (2.85–2.95)	0.10 (0.06–0.15)	0.276	Not available*	Not available*
>3	2.98 (2.96–3.00)	2.92 (2.88–2.95)	0.06 (0.02–0.10)		0.28 (0.11–0.71)	
Cause of renal failure						
DM	2.97 (2.92–3.01)	2.87 (2.80–2.95)	0.10 (0.01–0.18)	0.487	0.25 (0.06–1.13)	0.353
Other than DM	2.99 (2.98–3.00)	2.92 (2.89–2.95)	0.07 (0.04–0.11)		0.11 (0.04–0.34)	

Abbreviation: RMST, restricted mean survival time; RMTL, restricted mean time lost; LKT, living donor kidney transplantation; HD, hemodialysis; DM, diabetes mellitus.

* Ratio of RMTL could not be calculated because RMTL was zero in the LKT group.

Supplementary Table 4. Baseline characteristics before and after matching in the supplementary model.

	Before matching			After matching		
	LKT (N = 5,299)	HD (N = 208,145)	SMD	LKT (N = 3,160)	HD (N = 3,160)	SMD
Age (years)	48.8 ± 13.0	66.1 ± 12.4	1.36	49.3 ± 12.7	49.3 ± 12.7	0.00
Men (%)	64.2	61.8	0.05	63.0	63.0	0.00
Dialysis vintage (years)	2.3 (1.0–5.8)	5.5 (2.5–10.5)	0.52	2.6 (1.1–6.4)	2.7 (1.1–6.5)	0.00
Cause of renal failure (%)						
Glomerulonephritis	34.5	37.8	0.06	37.5	37.5	0.00
DM	20.4	35.6	0.34	19.4	19.4	0.00
Hypertension	4.4	7.2	0.12	3.9	3.9	0.00
Polycystic kidney disease	5.5	3.5	0.10	5.5	5.5	0.00
Others/unknown	35.2	16.2	0.45	33.7	33.7	0.00
Dialysis time (hour/week)		11.7 ± 1.8			11.7 ± 1.8	
BMI (kg/m^2)		21.2 ± 4.5			22.2 ± 4.2	
Kt/V		1.40 ± 0.30			1.35 ± 0.32	
nPCR (g/kg/day)		0.88 ± 0.18			0.90 ± 0.18	
Past history (%)						
Myocardial infarction		7.5			3.3	
Cerebral hemorrhage		4.7			2.7	
Cerebral infarction		14.7			5.1	
Amputation		2.9			1.0	
Hip fracture		2.9			0.7	

Supplementary Table 4. Continued

	Before matching			After matching		
	LKT (N = 5,299)	HD (N = 208,145)	SMD	LKT (N = 3,160)	HD (N = 3,160)	SMD
Laboratory test						
Creatinine (mg/dL)		10.2 ± 3.0			11.7 ± 3.2	
Hemoglobin (g/dl)		10.6 ± 1.3			10.7 ± 1.2	
Albumin (g/dL)		3.7 ± 0.4			3.9 ± 0.4	
Corrected calcium (mg/dL)		9.3 ± 0.9			9.2 ± 0.8	
Phosphorus (mg/dL)		5.1 ± 1.5			5.4 ± 1.6	
Intact PTH (pg/mL)		124 (63–208)			137 (74–234)	
CRP (mg/dL)		0.1 (0.0–0.4)			0.1 (0.0–0.2)	
Performance Status (%)						
Grade 0		45.1			71.1	
Grade 1		29.4			28.9	
Grade 2		12.9			0.0	
Grade 3		7.1			0.0	
Grade 4		5.5			0.0	
Dementia (%)						
No dementia		89.8			100.0	
Dementia not requiring care		4.5			0.0	
Dementia requiring care		5.7			0.0	

Abbreviations: LKT, living donor kidney transplantation; HD, hemodialysis; SMD, standardized mean difference; DM, diabetes mellitus; BMI, body mass index; nPCR, normalized protein catabolic rate; PTH, parathyroid hormone; CRP, C-reactive protein.

Supplementary Table 5. Sensitivity analysis in the supplementary model.

	Number of patients		HR (95% CI)
	LKT	HD	
Main analysis	3,160	3,160	0.38 (0.32–0.46)
Patients receiving LKT from 2009 to 2010	1,148	1,148	0.39 (0.30–0.52)
All patients receiving LKT from 2009 to 2017	5,051	5,051	0.39 (0.33–0.45)
Only HD patients with Grade 0 performance status	3,102	3,102	0.44 (0.37–0.53)
Propensity score matching	3,217	3,217	0.40 (0.34–0.48)
IPTW	3,281	151,074	0.40 (0.33–0.49)
Multivariable Cox proportional hazard model	3,281	151,074	0.39 (0.34–0.45)
Multivariable Cox proportional hazard model including all patients receiving LKT from 2009 to 2017	5,299	151,074	0.41 (0.36–0.47)

Abbreviations: LKT, living donor kidney transplantation; HD, hemodialysis; HR, hazard ratio; IPTW, inverse probability of treatment weighting.

Supplementary Table 6. The seven-year RMST differences and RMTL ratios in all patients and subgroups in the supplementary model.

	RMST (years)		Difference in RMST (years)	P for interaction	Ratio of RMTL	P for interaction
	LKT	HD				
Overall	6.78 (6.75–6.82)	6.40 (6.35–6.46)	0.38 (0.32–0.44)		0.37 (0.30–0.44)	
Sex						
Male	6.72 (6.67–6.77)	6.35 (6.28–6.42)	0.38 (0.29–0.46)	0.990	0.42 (0.34–0.52)	0.018
Female	6.88 (6.84–6.92)	6.50 (6.42–6.59)	0.38 (0.29–0.47)		0.24 (0.16–0.35)	
Age, yr						
<50	6.92 (6.89–6.95)	6.69 (6.63–6.75)	0.23 (0.17–0.30)	<0.001	0.25 (0.16–0.39)	0.073
>50	6.65 (6.59–6.71)	6.14 (6.05–6.22)	0.51 (0.41–0.62)		0.41 (0.33–0.50)	
Dialysis vintage, yr						
<3	6.86 (6.82–6.90)	6.45 (6.38–6.52)	0.41 (0.33–0.49)	0.529	0.26 (0.19–0.35)	0.012
>3	6.70 (6.63–6.76)	6.35 (6.27–6.43)	0.35 (0.25–0.45)		0.47 (0.37–0.59)	
Cause of renal failure						
DM	6.65 (6.55–6.75)	5.99 (5.84–6.14)	0.65 (0.47–0.83)	0.001	0.35 (0.26–0.48)	0.785
Other than DM	6.81 (6.78–6.85)	6.50 (6.45–6.56)	0.31 (0.25–0.38)		0.37 (0.30–0.47)	

Abbreviation: RMST, restricted mean survival time; RMTL, restricted mean time lost; LKT, living donor kidney transplantation; HD, hemodialysis; DM, diabetes mellitus.

Supplementary Table 7. The five-year RMST differences and RMTL ratios in all patients and subgroups in the supplementary model.

	RMST (years)		Difference in RMST (years)	P for interaction	Ratio of RMTL	P for interaction
	LKT	HD				
Overall	4.90 (4.88–4.92)	4.70 (4.67–4.73)	0.20 (0.16–0.24)		0.33 (0.26–0.42)	
Sex						
Male	4.87 (4.84–4.90)	4.68 (4.63–4.72)	0.20 (0.15–0.25)	0.823	0.39 (0.30–0.51)	0.023
Female	4.95 (4.93–4.97)	4.74 (4.69–4.80)	0.21 (0.15–0.26)		0.20 (0.12–0.33)	
Age, yr						
<50	4.96 (4.95–4.98)	4.84 (4.81–4.88)	0.12 (0.08–0.16)	<0.001	0.24 (0.15–0.40)	0.187
>50	4.85 (4.81–4.88)	4.57 (4.52–4.62)	0.28 (0.21–0.34)		0.36 (0.28–0.47)	
Dialysis vintage, yr						
<3	4.94 (4.92–4.96)	4.72 (4.68–4.77)	0.22 (0.17–0.26)	0.549	0.22 (0.15–0.33)	0.017
>3	4.86 (4.82–4.90)	4.67 (4.63–4.72)	0.18 (0.12–0.25)		0.44 (0.32–0.59)	
Cause of renal failure						
DM	4.86 (4.81–4.91)	4.49 (4.40–4.58)	0.37 (0.27–0.48)	0.001	0.27 (0.18–0.42)	0.435
Other than DM	4.91 (4.89–4.93)	4.75 (4.72–4.79)	0.16 (0.12–0.20)		0.36 (0.27–0.47)	

Abbreviation: RMST, restricted mean survival time; RMTL, restricted mean time lost; LKT, living donor kidney transplantation; HD, hemodialysis; DM, diabetes mellitus.

Supplementary Table 8. The three-year RMST differences and RMTL ratios in all patients and subgroups in the supplementary model.

	RMST (years)		Difference in RMST (years)	P for interaction	Ratio of RMTL	P for interaction
	LKT	HD				
Overall	2.96 (2.95–2.97)	2.90 (2.89–2.92)	0.06 (0.04–0.06)		0.41 (0.31–0.56)	
Sex						
Male	2.95 (2.93–2.96)	2.90 (2.88–2.92)	0.06 (0.03–0.07)	0.273	0.51 (0.37–0.72)	0.030
Female	2.98 (2.97–2.99)	2.91 (2.89–2.94)	0.07 (0.04–0.10)		0.21 (0.11–0.43)	
Age, yr						
<50	2.99 (2.98–3.00)	2.95 (2.93–2.96)	0.04 (0.02–0.06)	0.088	0.24 (0.12–0.48)	0.088
>50	2.93 (2.92–2.95)	2.86 (2.84–2.89)	0.07 (0.04–0.10)		0.48 (0.34–0.67)	
Dialysis vintage, yr						
<3	2.98 (2.96–2.99)	2.91 (2.89–2.93)	0.07 (0.05–0.09)	0.154	0.26 (0.16–0.43)	0.015
>3	2.94 (2.92–2.96)	2.90 (2.88–2.92)	0.04 (0.01–0.07)		0.58 (0.39–0.85)	
Cause of renal failure						
DM	2.95 (2.92–2.97)	2.83 (2.79–2.87)	0.12 (0.07–0.17)	0.009	0.32 (0.18–0.56)	0.272
Other than DM	2.96 (2.95–2.97)	2.92 (2.91–2.94)	0.04 (0.03–0.06)		0.46 (0.32–0.66)	

Abbreviation: RMST, restricted mean survival time; RMTL, restricted mean time lost; LKT, living donor kidney transplantation; HD, hemodialysis; DM, diabetes mellitus.
